# Pelvic injury prognosis is more closely related to vascular injury severity than anatomical fracture complexity: the WSES classification for pelvic trauma makes sense

**DOI:** 10.1186/s13017-020-00328-x

**Published:** 2020-08-17

**Authors:** Yu-Tung Wu, Chi-Tung Cheng, Yu-San Tee, Chih-Yuan Fu, Chien-Hung Liao, Chi-Hsun Hsieh

**Affiliations:** Department of Trauma and Emergency Surgery, Linkou branch of Chang Gung Memorial Hospital, 5, Fuxing St., Guishan Dist, Taoyuan City, 33305 Taiwan

**Keywords:** Pelvic fracture, Transfusion, Resuscitation, Length of hospital stay, Severity of injury

## Abstract

**Background:**

The most common cause of death in cases of pelvic trauma is exsanguination caused by associated injuries, not the pelvic injury itself. For patients with relatively isolated pelvic trauma, the impact of vascular injury severity on outcome remains unclear. We hypothesized that the severity of the pelvic vascular injury plays a more decisive role in outcome than fracture pattern complexity.

**Methods:**

Medical records of patients with pelvic fracture at a single center between January 2016 and December 2017 were retrospectively reviewed. Those with an abbreviated injury scale (AIS) score ≥ 3 in areas other than the pelvis were excluded. Lateral compression (LC) type 1 fractures and anteroposterior compression (APC) type 1 fractures according to the Young-Burgess classification and ischial fractures were defined as simple pelvic fractures, while other fracture types were considered complicated pelvic fractures. Based on CT, vascular injury severity was defined as minor (fracture with or without hematoma) or severe (hematoma with contrast pooling/extravasation). Patient demographics, clinical parameters, and outcome measures were compared between the groups.

**Results:**

Severe vascular injuries occurred in 26 of the 155 patients and were associated with poorer hemodynamics, a higher injury severity score (ISS), more blood transfusions, and a longer ICU stay (3.81 vs. 0.86 days, *p* = 0.000) and total hospital stay (20.7 vs. 10.1 days, *p* = 0.002) compared with minor vascular injuries. By contrast, those with complicated pelvic fractures (LC II/III, APC II/III, vertical shear, and combined type fracture) required a similar number of transfusions and had a similar length of ICU stay as those with simple pelvic fractures (LC I, APC I, and ischium fracture) but had a longer total hospital stay (13.6 vs. 10.3 days, *p* = 0.034). These findings were similar even if only patients with ISS ≥ 16 were considered.

**Conclusions:**

Our results indicate that even in patients with relatively isolated pelvic injuries, vascular injury severity is more closely correlated to the outcome than the type of anatomical fracture. Therefore, a more balanced classification of pelvic injury that takes both the fracture pattern and hemodynamic status into consideration, such as the WSES classification, seems to have better utility for clinical practice.

## Background

Pelvic fracture is one of the most complex injuries in trauma care. These patients are usually young and have a high overall injury severity score (ISS). The mortality rates remain high, particularly in those patients with hemodynamic instability and severe associated injuries [1–4].

To describe the severity of pelvic fracture, the Young-Burgess (YB) [5] and Tile [6] classification systems are the two most commonly recognized systems in the literature. The type of fracture in the YB system is based on the mechanism of injury, and the grade depends on the degree of ligamental disruption and pelvic instability [7]. The Tile system is based on the integrity of the sacroiliac ligament of the pelvis and its mechanical instability [6]. Although the anatomical fracture pattern is no doubt an important component determining the likelihood of significant vascular injury, the utility of the YB and Tile classification systems in predicting the need for blood transfusion and angiography in the initial resuscitation phase has shown mixed results [8–11]. There have been several studies supporting their predictability, but these results could not be consistently replicated across all of the studies [12–14].

On the other hand, the initial management of pelvic trauma focuses mainly on altered physiology and associated injuries and less on pelvic ring lesions [15]. Therefore, the priorities of pelvic fracture management are controlling bleeding, stabilizing hemodynamics, correcting coagulopathy, and treating associated injuries, followed by achieving definite stabilization of the pelvic ring [16]. Furthermore, evidence has also suggested that the most important predictor of mortality is the ISS, representing the totality of the injury, but not pelvic fracture instability [17].

From this point of view, since uncontrolled hemorrhaging remains a major cause of death in cases of pelvic fracture and the hemorrhage severity is not necessarily correlated with the fracture pattern, it appears that the status of pelvic exsanguination should be considered a much more important factor than that of pelvic ring disruption in predicting the outcome. We hypothesize that even in pelvic trauma patients with no or only minor injuries outside of the pelvis (in whom the pelvic ring fracture per se should reasonably be expected to play a more significant role in the outcome), the prognosis is more closely related to the severity of the pelvic vascular injury than the pelvic ring fracture.

## Methods

This was a retrospective case-cohort study approved by the Institutional Review Board of Chang Gung Memorial Hospital. From January 2016 to December 2017, 8111 trauma patients were registered in the Chang Gung Memorial Hospital trauma registry. Four hundred and twenty-five out of these 8111 patients had a pelvic fracture. The aim of the current study was to investigate the correlation of vascular injury and fracture pattern with the clinical outcome of trauma patients whose principle injury was pelvic fracture. Therefore, all patients with a diagnosis of pelvic fracture were included in the study if they were older than 18 years old and did not have an abbreviated injury scale (AIS) score higher than 2 in any body region other than the pelvis. As a result, a total of 155 patients were included in this study. All patients were treated by following a standardized protocol for initial resuscitation and management according to ATLS recommendations [18] and pelvic trauma treatment guidelines [19].

Their medical records were reviewed carefully, and data were collected regarding patient demographics and clinical profiles, including age, sex, mechanism of injury, hemodynamics upon ER admission, AIS score, ISS, type and grade of pelvic fracture, computed tomography (CT) findings regarding vascular injury and hemorrhage, number of blood transfusions during ER resuscitation and throughout hospitalization, length of ICU stay, total length of hospital stay, and mortality. In general, the algorithm for initial management was similar to the well-recognized guidelines in the literature [16, 19]: hemodynamic instability was defined by a systolic blood pressure less than 90 mmHg upon ER admission; CT was performed for all hemodynamically stable patients and for those hemodynamically unstable patients who could be stabilized after resuscitation; angioembolization was considered for those patients who showed a contrast blush on CT or those who showed no contrast blush on CT but still showed signs of ongoing bleeding [20].

The fracture pattern and severity of vascular injury were determined based on the CT results. The YB classification system [7] was used to determine the complexity of pelvic fracture. Lateral compression (LC) type I, anteroposterior compression (APC) type I, and ischial fractures were classified as stable pelvic fractures (simple pelvic fractures, s-PFs), while LC type II and III, APC type II and III, vertical shearing (VS), and combined type fractures were classified as unstable pelvic fractures (complicated pelvic fractures, c-PFs). In addition, the severity of vascular injury was recorded as minor (pelvic fracture without retroperitoneal hematoma or hematoma without contrast blush) or severe (hematoma with contrast pooling or extravasation).

Each patient was assigned to one of the groups according to the complexity of pelvic fracture (s-PF or c-PF) and severity of vascular injury (minor or severe). The patient demographics, clinical parameters, and outcome measures were compared between the groups.

As severe vascular injury is more likely to occur in cases of complicated pelvic fractures, those patients with an ISS ≥ 16 were selected for further analysis to clarify the relative importance of vascular injury and pelvic ring fracture in these cases of severe trauma. There were 86 patients who had an ISS ≥ 16 in the current study.

In 2017, the World Society of Emergency Surgery (WSES) published its guidelines for the classification and management of pelvic trauma. The WSES classification takes both the pelvic fracture pattern and hemodynamic stability into consideration. To verify the hypothesis of the current study that the prognosis of pelvic trauma is more closely related to the severity of the pelvic vascular injury than the pelvic ring fracture, the patients’ pelvic injuries were classified as mild, moderate, or severe according to the WSES classification, and patient demographics, clinical parameters, and outcome measures were compared between the groups.

Descriptive statistics were calculated for the cohort. Frequency tables were generated for categorical variables, and continuous variables are summarized by the mean and standard deviation (SD). Continuous data were analyzed using Student’s *t* test or one-way ANOVA to compare the means of two or more independent groups, respectively. Tukey’s post hoc test was used following one-way ANOVA to test for differences between the groups. All statistical analyses were performed using the SPSS computer software package (version 21.0, Chicago, IL, USA). A value of *p* < 0.05 was considered to be statistically significant.

## Results

Overall, among the 155 patients included in the study, there were 71 (45.8%) males and 84 (54.2%) females, with a mean age of 44.7 ± 21 years. The majority of the patients (*n* = 117, 75.5%) were involved in a traffic accident, while 19 (12.3%) patients were injured due to a slip, 11 (7.1%) patients were injured by a fall, and 8 (5.2%) patients were crushed/rolled over by heavy objects or machines. There were 79 patients with s-PFs and 76 patients with c-PFs. On the other hand, according to the abovementioned definitions, 129 patients had minor vascular injuries, and the other 26 patients had severe vascular injuries. The mean ISS was 14 ± 4.9, and the mean length of ICU stay and total length of hospital stay was 1.4 ± 3.9 and 11.8 ± 9.8 days, respectively (Table 1). The same clinical profiles and outcome measures were analyzed for those patients with an ISS ≥ 16, as shown in Table 1.

**Table 1:** Patient demographics

	All patients	Patients with ISS ≥ 16
*n*	155	86
Age (years)	44.7 ± 21.4	44.1 ± 21.9
Sex (M/F)	71/84	37/49
SBP (mmHg)	125.7 ± 28.0	121.8 ± 29.2
HR (/min)	93.2 ± 17.9	95.7 ± 19.8
ISS	14.0 ± 4.9	17.7 ± 2.9
RTS	7.73 ± 0.4	7.68 ± 0.4
TRISS	0.97 ± 0.03	0.96 ± 0.04
Fracture type		
Simple fracture		
*LC1*	65 (41.9%)	30 (34.8%)
*APC1*	5 (3.2%)	3 (3.5%)
*Ischial fracture*	9 (5.8%)	0 (0%)
Complicated fracture		
*LC2*	41 (25.5%)	24 (27.9%)
*LC3*	5 (3.2%)	5 (5.8%)
*APC2*	6 (3.9%)	6 (7.0%)
*APC3*	0 (0%)	0 (0%)
*VS*	3 (1.9%)	3 (3.5%)
*Combined*	21 (13.5%)	15 (17.4%)
CT findings		
Mild vascular injury		
*No hematoma*	38 (24.5%)	1 (1.2%)
*Hematoma without contrast pooling*	91 (58.7%)	60 (69.7%)
Severe vascular injury		
*Hematoma with contrast pooling*	26 (26.7%)	25 (29.0%)
Mortality	0%	0%
ICU LOS (days)	1.4 ± 3.9	1.65 ± 3.0
Hospital LOS (days)	11.8 ± 9.8	13.8 ± 10.6

*SBP* systolic blood pressure, *HR* heart rate, *ISS* injury severity score, *RTS* revised trauma score, *TRISS* trauma injury severity score, *LC1/LC2/LC3* lateral compression type 1/2/3, *APC1/APC2/APC3* anteroposterior compression type 1/2/3, *VS* vertical shear, *LOS* length of stay

Patients with either an s-PF or a c-PF were similar in age and the mean pulse rate and systolic blood pressure at the time of hospital arrival. The ISS was significantly higher in the c-PF group, and the total length of hospital stay was longer; however, there was no difference regarding the number of transfusions required or the length of ICU stay (Table 2). However, for those patients with an ISS ≥ 16, there were no differences in any of the analyzed parameters or outcome measures (Table 2).

**Table 2:** Comparison of clinical parameters and outcomes between patients with a simple or complicated pelvic ring fracture

	All patients (*n* = 155)			Patients with ISS ≥ 16 (*n* = 86)		
	Simple PF	Complicated PF	*p*	Simple PF	Complicated PF	*p*
No. of patients	79	76		34	52	
Age (years)	46.1 ± 22.5	43.2 ± 20.3	0.403	45.6 ± 25.3	43.2 ± 21.3	0.639
SBP (mmHg)	127.8 ± 25.6	123.4 ± 30.2	0.323	123.2 ± 22.4	120.8 ± 33.0	0.683
HR (bpm)	92.7 ± 18.4	93.8 ± 17.3	0.701	95.7 ± 21.7	95.6 ± 18.7	0.983
ISS	12.8 ± 4.7	15.2 ± 4.8	0.002*	17.4 ± 2.5	17.9 ± 3.2	0.484
RTS	7.73 ± 0.45	7.71 ± 0.35	0.846	7.71 ± 0.48	7.66 ± 0.41	0.601
TRISS	0.97 ± 0.04	0.97 ± 0.02	0.918	0.95 ± 0.05	0.96 ± 0.02	0.424
ICU LOS (days)	1.15 ± 4.3	1.57 ± 3.4	0.512	1.24 ± 2.04	1.92 ± 3.50	0.304
Hospital LOS (days)	10.27 ± 9.2	13.6 ± 10.1	0.034*	12.0 ± 8.5	14.4 ± 11.7	0.310
Transfusion (unit)						
ER pRBC	0.63 ± 2.3	0.83 ± 1.9	0.567	0.94 ± 2.7	1.15 ± 2.2	0.696
ER FFP	0.45 ± 1.8	0.81 ± 2.4	0.295	0.64 ± 1.8	1.15 ± 2.8	0.362
ER PLT	0.30 ± 1.9	0.31 ± 1.9	0.969	0.35 ± 2.0	0.46 ± 2.3	0.826
Total pRBC	4.41 ± 14.5	6.0 ± 7.3	0.396	4.73 ± 8.6	7.38 ± 8.3	0.153
Total FFP	2.83 ± 11.9	3.67 ± 7.5	0.605	3.29 ± 10.4	4.71 ± 8.5	0.493
Total PLT	3.34 ± 14.5	3.16 ± 10.9	0.929	4.94 ± 14.8	4.38 ± 13.0	0.855

*SBP* systolic blood pressure, *HR* heart rate, *bpm* beats per minute, *ISS* injury severity score, *RTS* revised trauma score, *TRISS* trauma injury severity score, *LOS* length of stay, *ER pRBC/FFP/PLT* units of packed red blood cells/fresh-frozen plasma/platelets transfused in the emergency room; *Total pRBC/FFP/PLT* units of packed red blood cells/fresh-frozen plasma/platelets transfused throughout hospitalization. *p < 0.05 with statistical significance

In contrast, regarding the clinical parameters and outcome measures, a number of differences were noted between patients with minor and severe vascular injuries. Those who sustained a severe vascular injury had a significantly increased heart rate upon hospital arrival, a higher ISS and revised trauma score (RTS), and a significantly lower trauma injury severity score (TRISS). The number of transfusions was larger both in the ER resuscitation phase and throughout hospitalization. Finally, the length of ICU stay and the total length of hospital stay were significantly longer in those with severe vascular injuries than in those with minor vascular injuries (Table 3).

**Table 3:** Comparison of clinical parameters and outcomes between patients with minor or major pelvic vascular injury

	All patients (*n* = 155)			Patients with ISS ≥ 16 (*n* = 86)		
	Minor vascular injury	Major vascular injury	*p*	Minor vascular injury	Major vascular injury	*p*
No. of patients	129	26		61	25	
Age (years)	44.6 ± 21.0	44.8 ± 23.9	0.977	43.9 ± 22.4	44.6 ± 24.4	0.905
SBP (mmHg)	127.3 ± 25.8	117.7 ± 36.5	0.214	123.1 ± 25.5	118.5 ± 37.1	0.575
HR (bpm)	91.6 ± 16.3	101.2 ± 22.9	0.049*	93.9 ± 18.5	100.0 ± 22.5	0.193
ISS	13.1 ± 4.4	18.6 ± 4.8	0.000*	17.2 ± 1.9	19.0 ± 4.5	0.061
RTS	7.77 ± 0.36	7.50 ± 0.54	0.024*	7.76 ± 0.36	7.49 ± 0.54	0.031*
TRISS	0.97 ± 0.03	0.95 ± 0.03	0.005*	0.96 ± 0.04	0.95 ± 0.03	0.205
ICU LOS (days)	0.86 ± 3.78	3.81 ± 3.66	0.000*	0.95 ± 2.78	3.36 ± 2.92	0.001*
Hospital LOS (days)	10.13 ± 7.1	20.73 ± 15.2	0.002*	10.62 ± 6.0	20.44 ± 15.4	0.005*
Transfusion (unit)						
ER pRBC	0.37 ± 1.4	2.50 ± 3.6	0.006*	0.46 ± 1.3	2.56 ± 3.8	0.010*
ER FFP	0.21 ± 1.2	2.69 ± 3.8	0.003*	0.23 ± 1.0	2.72 ± 3.9	0.004*
ER PLT	0.09 ± 1.0	1.38 ± 3.9	0.107	0	1.44 ± 3.9	0.083
Total pRBC	3.82 ± 11.3	12.0 ± 10.5	0.001*	4.11 ± 6.0	11.8 ± 10.7	0.002*
Total FFP	2.31 ± 9.8	7.84 ± 9.7	0.012*	2.80 ± 8.9	7.44 ± 9.7	0.035*
Total PLT	2.14 ± 11.7	8.76 ± 16.4	0.060	2.95 ± 11.9	8.64 ± 16.8	0.132

*SBP* systolic blood pressure, *HR* heart rate, *bpm* beats per minute, *ISS* injury severity score, *RTS* revised trauma score, *TRISS* trauma injury severity score, *LOS* length of stay, *ER pRBC/FFP/PLT* units of packed red blood cells/fresh-frozen plasma/platelets transfused in the emergency room, *Total pRBC/FFP/PLT* units of packed red blood cells/fresh-frozen plasma/platelets transfused throughout hospitalization. *p < 0.05 with statistical significance

Among patients with an ISS ≥ 16, significant differences were also noted between those with minor and severe vascular injuries. Those who sustained a severe vascular injury had a significantly lower RTS, required a larger number of transfusions both in the ER resuscitation phase and throughout hospitalization, and had longer stays in the ICU and hospital (Table 3).

**Fig. 1:**
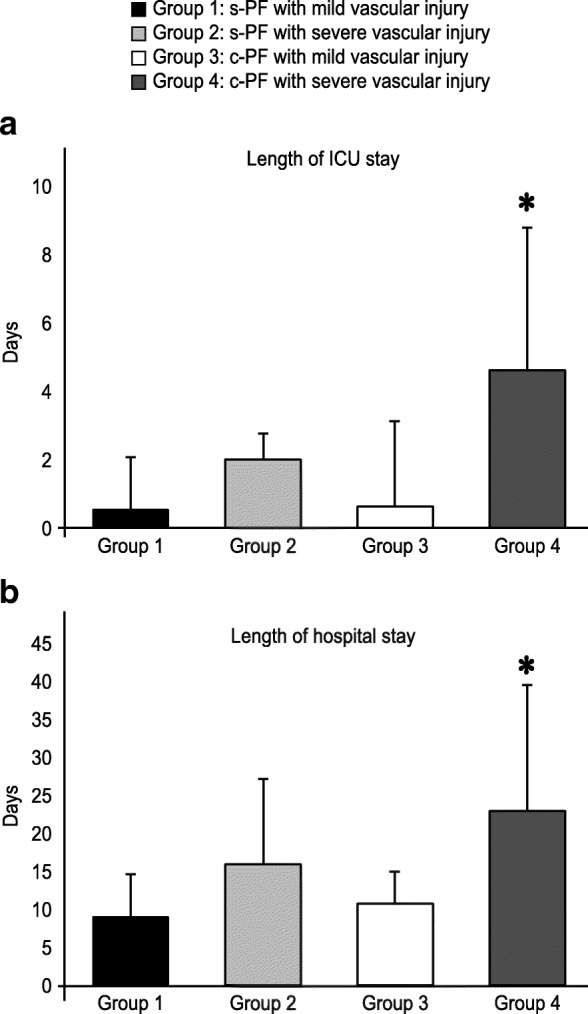
Length of hospital stay in patients with pelvic injury. Comparison of the mean length of **a** ICU stay and **b** total hospital stay among patients with simple or complicated pelvic fracture with mild or severe vascular injury. Data are shown as the mean ± standard deviation. *p < 0.05 compared to groups 1, 2, and 3. s-PF simple pelvic fracture, c-PF complicated pelvic fracture

**Fig. 2:**
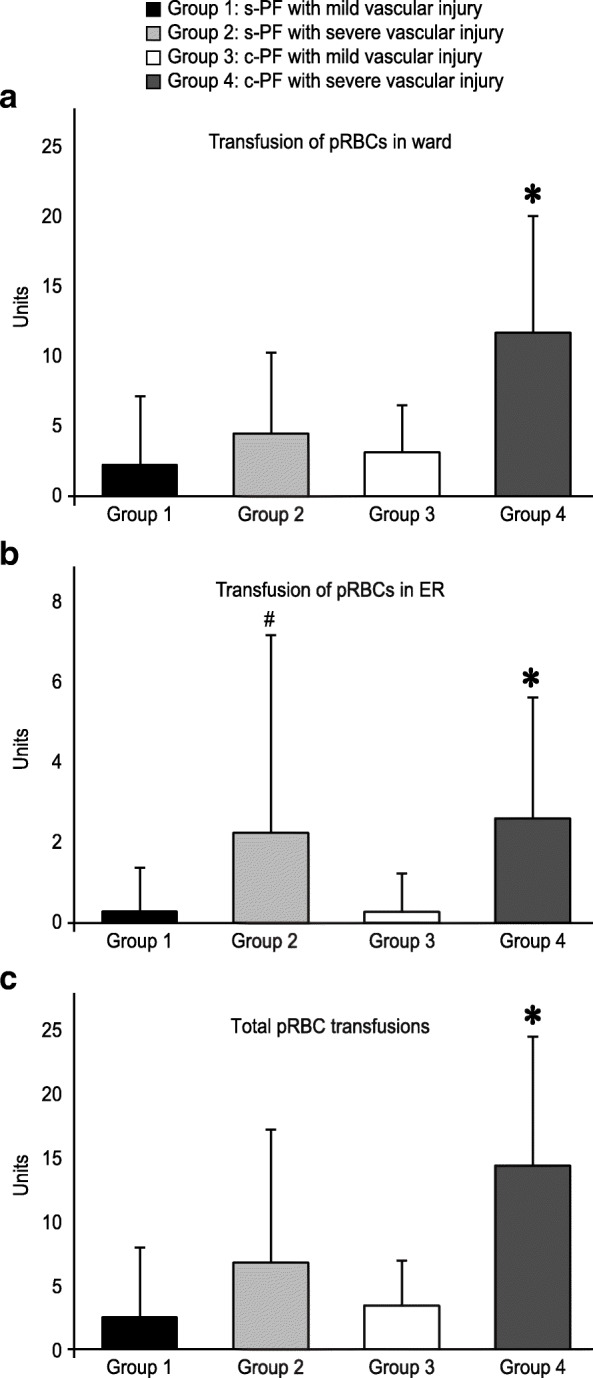
Number of blood transfusions in patients with pelvic injury. Comparison of the mean number of packed red blood cell (pRBC) units transfused **a** after admission to the ward, **b** during ER resuscitation, and **c** throughout hospitalization among patients with simple or complicated pelvic fracture with mild or severe vascular injury. Data are shown as the mean ± standard deviation. *p < 0.05 compared to groups 1, 2, and 3; ^#^*p* < 0.05 compared to groups 1 and 3

All of the patients were divided into 4 groups according to the pattern of pelvic fracture and severity of vascular injury, as follows: group 1: simple pelvic fracture with mild vascular injury; group 2: simple fracture with severe vascular injury; group 3: complicated fracture with mild vascular injury; and group 4: complicated fracture with severe vascular injury. Patients in group 4 had significantly longer stays in the ICU and hospital than patients in all of the other groups (Fig. 1). Furthermore, patients in group 4 required a significantly larger number of transfusions than patients in the other groups not only during the resuscitation stage in the ER but also throughout hospitalization (Fig. 2). In contrast, the transfusion requirement for patients in group 2 (simple fracture with severe vascular injury) was significantly higher than that for patients in group 3 (complicated fracture with mild vascular injury) during the ER resuscitation stage, but the requirements were similar during the remaining period of hospitalization (Fig. 2).

By dividing the patients into mild, moderate, and severe pelvic injuries according to the WSES classification for pelvic trauma, our results showed that admission hemodynamics, RTS, ISS, TRISS, length of ICU and total hospital stay, and amount of blood transfusions were all similar between the patients with mild and moderate pelvic injuries. In contrast, patients with severe pelvic injuries had significantly lower systolic blood pressure and increased heart rate on hospital arrival, significantly higher ISS, significantly lower RTS and TRISS, significantly prolonged length of ICU and total hospital stay, and significantly larger amounts of blood transfusions than patients with mild and moderate pelvic injuries (Table 4).

**Table 4:** Comparison of patients with minor, moderate, and severe pelvic injuries according to the WSES classification

	WSES pelvic injury classification					
	Minor (Gr. A)	Moderate (Gr. B)	Severe (Gr. C)	*p* (Gr. A vs. B)	*p* (Gr A vs. C)	*p* (Gr. B vs. C)
No. of patients	72	66	17			
Age (years)	46.4 ± 21.8	43.8 ± 20.8	40.5 ± 23.3	0.76	0.58	0.84
SBP (mmHg)	131.1 ± 24.1	130.2 ± 26.0	84.0 ± 15.2	0.97	0.00*	0.00*
HR (bpm)	90.5 ± 16.8	93.4 ± 17.2	103.4 ± 21.4	0.59	0.02*	0.10
ISS	12.8 ± 4.8	14.6 ± 4.5	16.5 ± 5.7	0.07	0.01*	0.34
RTS	7.82 ± 0.11	7.81 ± 0.13	7.02 ± 0.69	1.0	0.00*	0.00*
TRISS	0.97 ± 0.21	0.97 ± 0.23	0.94 ± 0.08	0.94	0.00*	0.00*
ICU LOS (days)	0.71 ± 1.56	1.06 ± 2.69	3.25 ± 4.79	0.69	0.00*	0.01*
Hospital LOS (days)	9.72 ± 6.9	11.7 ± 5.4	19.1 ± 18.8	0.34	0.00*	0.01*
Transfusion (unit)						
ER pRBC	0.38 ± 1.8	0.63 ± 1.7	1.93 ± 2.5	0.72	0.01*	0.04*
ER FFP	0.19 ± 1.0	0.42 ± 1.8	2.75 ± 3.6	0.73	0.00*	0.00*
ER PLT	0.16 ± 1.4	0.18 ± 1.4	0.75 ± 3.0	0.99	0.41	0.44
Total pRBC	2.86 ± 6.2	4.87 ± 6.3	9.81 ± 9.3	0.18	0.00*	0.02*
Total FFP	1.69 ± 7.3	3.01 ± 7.1	5.87 ± 7.7	0.53	0.09	0.33
Total PLT	2.3 ± 10.9	2.54 ± 10.6	4.50 ± 10.6	0.99	0.73	0.78

*SBP* systolic blood pressure, *HR* heart rate, *bpm* beats per minute, *ISS* injury severity score, *RTS* revised trauma score, *TRISS* trauma injury severity score, *LOS* length of stay, *ER pRBC/FFP/PLT* units of packed red blood cells/fresh-frozen plasma/platelets transfused in the emergency room; *Total pRBC/FFP/PLT* units of packed red blood cells/fresh-frozen plasma/platelets transfused throughout hospitalization. *p < 0.05 with statistical significance

## Discussion

Managing pelvic injuries continues to be a challenge for even the most experienced trauma surgeons. Pelvic fractures frequently result from a high-energy impact and are usually associated with multisystem injuries and catastrophic hemorrhage. As reported by Lunsjo et al. [17] and Agri et al. [21], most deaths related to pelvic fracture were caused by associated injuries, not the pelvic fracture itself. In these patients, the most common cause of death was severe traumatic brain injury [17, 21, 22]. Therefore, to specifically investigate the correlation of the fracture pattern and pelvic vascular injury severity with the outcome, patients with an AIS score higher than 2 for body regions other than the pelvis were excluded from the current study.

By dividing the patients into the s-PF and c-PF groups according to the fracture pattern, our results reveal that although the fracture complexity correlated well with the length of hospital stay, it had a nonsignificant correlation with the number of transfusions required. In an earlier study by Poole et al. [23], although the injury severity was correlated with the pelvic fracture severity, hospital outcomes were determined by associated injuries and not by the pelvic fracture. Furthermore, systems for the classification of pelvic injuries based on pelvic ring stability and their relevance to the association with transfusion requirements and mortality have been disputed in the literature. Osterhoff et al. [10] reported the value of the Tile and YB classification systems in predicting mortality, transfusion requirements, and concomitant injuries. The number of transfusions significantly increased with increasing fracture pattern severity [10]. Similarly, Manson et al. [11] reported that patients with an unstable pelvic fracture based on the YB classification had higher transfusion requirements than those with a stable fracture. Nonetheless, one should note that in both Osterhoff’s and Manson’s studies, patients with severe pelvic fractures were more likely to have concomitant injuries that would lead to greater transfusion requirements. In contrast, an important difference between the current study and these two studies is that patients with significant concomitant injuries (AIS score > 2) were not included in the current study. Therefore, in the current study, patient hemorrhage was mainly caused by pelvic injuries. Under these conditions, our results show that the fracture pattern (simple or complicated) was not correlated with the number of transfusions. Our results are in line with those reported by Sarin et al. [13]. They found that the pelvic fracture pattern (with or without major ligamentous disruption) did not consistently correlate with the need for urgent embolization. This suggests that the risk of exsanguination or the need for transfusion due to complicated pelvic fracture is probably similar to that due to simple pelvic fracture.

Vascular injuries caused by pelvic fracture are life-threatening because they often present as multifocal, noncompressible arterial and venous hemorrhages. Tien et al. [24] analyzed 558 consecutive trauma deaths at their institution and found that the most common preventable cause of death was hemorrhage from blunt pelvic injuries. An incorrect choice of where to transport these patients for further intervention could delay the time to definite hemorrhage control and increase the risk of mortality. In this regard, the assessment of potential severe vascular injury and timely hemorrhage control should be the highest priorities in the acute management of pelvic fracture [25].

Our data show that the severity of vascular injury was significantly correlated with patient outcomes. Compared to patients with mild vascular injuries, patients with severe vascular injuries were more likely to have unstable hemodynamics; a higher ISS, RTS, and TRISS; a larger number of transfusions; and longer ICU and hospital stays. Consistent with our results, in a study that investigated the relationship of the hemorrhage volume with the outcome of pelvic fracture, Blackmore et al. [26] showed that subjects with large pelvic hemorrhage volumes were more likely to have pelvic arterial injuries and require a large number of transfusions. They also demonstrated a strong association between the pelvic hemorrhage volume and adverse clinical outcomes even though the pelvic fracture pattern was not taken into consideration in their study. Therefore, our results suggest that even for those patients with major injuries limited to the pelvic cavity, the severity of pelvic vascular injury appeared to be a much more significant factor than the pelvic fracture pattern in determining patient outcomes.

In addition, the above findings were still true even if only those patients with an ISS ≥ 16 were considered. According to the current AIS scoring system for pelvic fractures, the AIS score is 4 for a moderate pelvic hematoma with an estimated blood loss ≤ 20% by volume, while it is 5 for a large hematoma with an estimated blood loss volume ≥ 20% [27]. That is, a pelvic injury with the same fracture pattern would be given a different AIS score according to the size of the hematoma or the volume of blood loss. Regardless of the pattern of pelvic fracture, our patients would have an AIS score ≥ 4 as long as there was a significant amount of pelvic injury-related bleeding. Therefore, for patients with an ISS ≥ 16 (which most likely indicated the presence of severe vascular injury rather than a complicated pelvic ring fracture), it was not surprising that the severity of vascular injury was more prognostic in predicting patient outcomes than the complexity of pelvic ring fracture.

In 2017, the World Society of Emergency Surgery (WSES) published its guidelines for the classification and management of pelvic trauma [16]. The WSES guidelines emphasize that the optimal treatment strategy should be determined by the hemodynamic status and associated injuries in addition to anatomical lesions. The first decisions are based mainly on the hemodynamic conditions rather than on the pelvic ring lesions. According to the WSES classification, minor pelvic injuries are defined as those with mechanically stable (LC type I, APC type I) and hemodynamically stable lesions, while moderate injuries comprise those with mechanically unstable fracture (LC type II–III, APC type II–III, VS, and combined type fractures) but hemodynamically stable lesions. In addition, severe pelvic injuries are defined as hemodynamically unstable lesions independent of mechanical status. While the main differences between minor and moderate injuries are the complexity of the fracture patterns, any fractures that are associated with unstable hemodynamics are categorized as severe injuries. Our results showed that patient outcomes were similar between the WSES mild and moderate types of pelvic injuries; however, patients with severe pelvic injuries had significantly worse outcomes than those with the other two types of injuries. Therefore, hemodynamic instability appears to be a more relevant factor than the complexity of the fracture pattern for patient outcome. These results suggest that the WSES classification, which takes both the fracture pattern and hemodynamic status into consideration, might be clinically more useful than the classic YB classification system.

There were no cases of mortality in our series. The most critical factor of this result was that interventional radiologists were available at our institution for 24 h along with trauma surgeons. Most exsanguinating patients could be stabilized by transarterial embolization shortly after initial resuscitation whenever indicated [20, 25, 27, 28]. Another reason for the lack of mortality was that pelvic trauma patients with associated injuries that were confirmed to be the principal cause of death, such as severe brain injury, were not included in the current study [17, 21–23].

There are several limitations to this study. First, this was a single-center experience with relatively uniform practices based on standardized, acceptable guidelines. Second, given its retrospective nature, information bias and documentation errors in the trauma registry and medical records could have affected the accuracy of the data. Third, the findings of our study specifically came from a group of patients with pelvic trauma as the principle injury. As reported by Vaidya et al. [29], the leading cause of death from blunt pelvic trauma within 6 h of injury was hemorrhage from multiple areas but rarely from the pelvic injury alone; moreover, that between 6 and 24 h was severe head injury. Because patients with severe associated injuries were not a part of this cohort due to the design of the current study, outcome measures such as the number of transfusions, length of stay, and mortality should be interpreted with care when compared with the findings of other studies that included patients with multisystem trauma.

## Conclusion

This study compared the impact of the anatomical pelvic fracture pattern and severity of pelvic vascular injury on the outcomes of patients with only a relatively isolated pelvic injury. The severity of vascular injury was a more significant factor in determining patient outcomes than the fracture pattern. Therefore, a more balanced classification of pelvic injury that takes both the fracture pattern and hemodynamic status into consideration, such as the WSES classification, seems to have better utility for clinical practice than the classical YB classification system.

## Data Availability

The datasets used and/or analyzed during the current study are available from the corresponding author on reasonable request.

## Abbreviations

AIS: Abbreviated injury scale
ATLS: Advanced Trauma Life Support
c-PF: Complicated pelvic fracture
ER: Emergency room
ICU: Intensive care unit
ISS: Injury severity score
LOS: Length of stay
RTS: Revised trauma score
SBP: Systolic blood pressure
s-PF: Simple pelvic fracture
TRISS: Trauma injury severity score
